# MTS-Stega: Linguistic Steganography Based on Multi-Time-Step

**DOI:** 10.3390/e24050585

**Published:** 2022-04-22

**Authors:** Long Yu, Yuliang Lu, Xuehu Yan, Yongqiang Yu

**Affiliations:** 1 College of Electronic Engineering, National University of Defense Technology, Hefei 230037, China; yl@nudt.edu.cn (L.Y.); yy_qiang@nudt.edu.cn (Y.Y.); 2 Anhui Province Key Laboratory of Cyberspace Security Situation Awareness and Evaluation, Hefei 230037, China

**Keywords:** linguistic steganography, text generation, multi-time-step, fixed-length coding, imperceptibility, decoding efficiency

## Abstract

Generative linguistic steganography encodes candidate words with conditional probability when generating text by language model, and then, it selects the corresponding candidate words to output according to the confidential message to be embedded, thereby generating steganographic text. The encoding techniques currently used in generative text steganography fall into two categories: fixed-length coding and variable-length coding. Because of the simplicity of coding and decoding and the small computational overhead, fixed-length coding is more suitable for resource-constrained environments. However, the conventional text steganography mode selects and outputs a word at one time step, which is highly susceptible to the influence of confidential information and thus may select words that do not match the statistical distribution of the training text, reducing the quality and concealment of the generated text. In this paper, we inherit the decoding advantages of fixed-length coding, focus on solving the problems of existing steganography methods, and propose a multi-time-step-based steganography method, which integrates multiple time steps to select words that can carry secret information and fit the statistical distribution, thus effectively improving the text quality. In the experimental part, we choose the GPT-2 language model to generate the text, and both theoretical analysis and experiments prove the effectiveness of the proposed scheme.

## 1. Introduction

Shannon [1] summarized three basic information security systems, namely encryption system, privacy system and concealment system. The primary purpose of the encryption system is to protect the security of confidential information itself and make the message indecipherable through the key. The privacy system is designed to control access to confidential information so that non-authorized users cannot access important information. Both of these systems expose the existence of confidential information, making it vulnerable to attacks. Concealment system hides the confidential information into different carriers and transmits it through open channels, which can effectively hide the existence of confidential information and thus enhance its security.

Steganography is a crucial technique in concealment systems, which focuses on how to embed secret information into carriers efficiently and securely. Depending on the type of carrier [2], steganography can be divided into image steganography [3], text steganography [4], audio steganography [5] and video steganography [6]. The advantages of text over other digital carriers such as image and audio are: (1) Text is the main form of information communication for people, and its wide and universal usage scenarios give text steganography a broad application prospect. (2) Text has strong robustness when transmitted in public channels. Other carriers, such as digital images, usually produce a certain degree of distortion due to compression strategies when transmitted using public network channels, which may destroy confidential information contained therein. On the other hand, text is highly resistant to interference as it is transmitted in a public network environment with little to no information loss due to channel noise.

Bennett [7] summarized two strategies of linguistic steganography: modification-based steganography and generation-based steganography. Modification-based linguistic steganography mainly involves the synonymous substitution of semantic units in the text to embed confidential messages. For example, a synonym dictionary can be constructed, and then words in the dictionary can be encoded to embed information by replacing synonyms in the carrier text [8]. Alternatively, the syntactic structure can be equivalently transformed to represent different secret information [9]. However, the embedding capacity of such methods is relatively low, and it is difficult to convey a large amount of information. Moreover, due to the low redundancy of text itself, performing substitution operations is likely to lead to syntactic and semantic unnaturalness [10,11,12]. Generation-based linguistic steganography is to automatically generate a piece of text using language model (LM), encode the semantic units of the text during the generation process and select the output of the corresponding semantic units according to the confidential message to be embedded, so as to achieve steganographic information embedding. This strategy does not require prior preparation of the carrier but automatically generates the carrier containing the confidential message, so the steganographer has more freedom in the process of embedding the information and thus can expect to obtain a high rate of information embedding [13,14,15].

Currently, generation-based text steganography can be divided into two main categories, fixed-length coding-based steganography schemes [14,16,17] and variable-length coding-based steganography schemes [16,18,19,20,21,22,23]. Fang et al. [14] firstly split the word space into blocks, with several words in each block, and encoded the blocks using fixed-length coding. In the process of generating text, the corresponding block is determined according to the secret bitstream, from which suitable words are selected for output, thus completing the embedding of secret information. However, to adjust the information-embedding rate, all words in the entire dictionary need to be recombined and encoded, and the quality of the generated text decreases rapidly as the embedding rate slowly increases. Yang et al. [16] proposed fix-length coding (FLC) based on perfect binary tree and variable-length coding (VLC) based on Huffman tree. They encode the Top-K words in the candidate pool (CP) predicted by the language model at each time step according to the conditional probability and select the corresponding word for output according to the secret message, thereby generating the steganographic text. Xiang et al. [17] modeled natural sentences as letter sequences, used the Char-RNN model to obtain letter-level conditional probability distributions, and then encoded letters based on fixed-length coding. Many subsequent works based on variable-length coding followed Yang’s framework. They use arithmetic coding [18], STC coding [22], etc., to encode candidate words, and then select the corresponding words according to the secret message. Dai et al. [21] proposed patient-Huffman coding, which changed the construction of candidate pools based on Top-K to dynamic candidate pool construction.

The information-encoding efficiency of variable-length coding is lower than that of fixed-length coding, and the embedding rate of the latter is larger than that of the former for the same size of candidate pool (CPS). Moreover, fixed-length coding is simpler and has less computational overhead. In many cases, steganographic receivers do not have high-performance devices to extract secret information, and they may only have handheld or embedded devices to process steganographic text. In this scenario, high time efficiency and low computational complexity are required. The decoding advantage of fixed-length coding is particularly important in this resource-constrained environments, such as when miniaturization and lightweighting of the decoding side are required. Therefore, generative text steganography based on fixed-length coding deserves further research.

The steganographic schemes mentioned above determine a word according to the secret message to be embedded in one time step, and the choice of the word has no flexibility. The secret message has a great influence on the text generation, which may cause the selected words not to conform to the statistical distribution of the training text, thus reducing the concealment of the steganographic text. Therefore, how to reduce the influence of secret messages on candidate word selection in the process of steganography, so as to generate more natural text, is an urgent problem to be solved.

In the paper, we propose a text steganography method based on multi-time-step (MTS-stega), which integrates multiple time steps in the text generation process and selects the optimal multiple consecutive words to jointly carry a unit of secret message by solving the goal programming model. The contributions of this research are three-fold, as follows:

- For the problem that the selection of candidate words in current steganography methods is seriously restricted by secret message, which in turn affects the quality of steganographic text, we propose a multi-time-step method, which effectively reduces the impact of secret information on candidate word selection, thereby effectively improving the quality and imperceptibility of steganographic text.
- For the scenario where the resources of the steganographic receiver are limited and only handheld devices or embedded devices are used, we propose to use fixed-length coding to complete the mapping of word space to secret messages, which can effectively reduce the decoding complexity and improve information extraction efficiency compared to variable-length coding at the decoding end.
- We compare with existing fixed-length coding schemes in terms of the quality of generated text, and compare with mainstream variable-length coding schemes in terms of decoding efficiency. The experimental results demonstrate the effectiveness of this scheme.

The rest of this paper is organized as follows. Preliminaries and prior work are provided in Section 2. In Section 3, we will describe the architecture of our proposed method, including the information-hiding algorithm and information extraction algorithm. In Section 4, we will present the experimental setup and show the performance of the proposed method. The conclusions are summarized in Section 5.

## 2. Preliminaries and Prior Work

In this section, we first introduce the concept of the language model and evaluation metrics for generative text steganography, including perplexity and embedding rate. After that, we introduce the linguistic steganography based on FLC and explain its shortcomings as the goal of our solution.

The main notations used in this paper are as shown in Table 1:

### 2.1. Evaluation Metrics of Generation-Based Linguistic Steganography

In the field of natural language processing, text is usually considered as a sequence of words consisting of specific words combined according to semantic associations and syntactic rules, and a language model is used to describe the joint probability distribution of word sequences, whose expression is:

(1) $$\begin{array}{cc} P(S) & =P(W_{1},W_{2},\ldots ,W_{n})\\ \; & =P\left(W_{1}\right)P\left(W_{2}|W_{1}\right)\cdots P\left(W_{n}|W_{1}W_{2}\cdots W_{n-1}\right)\\ \; & =\prod_{1}^{n}P(W_{i}\left|W_{1}W_{2}\cdots W_{i-1}\right) \end{array}$$

where P(S) represents the generation probability of the word sequence $S=W_{1},W_{2},\ldots$, $W_{n}$, and $P(W_{n}|W_{1}W_{2}\cdots W_{n-1})$ denotes the conditional probability of generating word $W_{n}$ given $W_{1}W_{2}\cdots W_{n-1}$ above. The conditional probability reflects the degree of fit between the candidate word $W_{n}$ and the previous text. The higher the conditional probability, the more reasonable the generated text. Due to the diversity of language expressions, there are usually multiple candidate words $W_{n}$ for a given above $W_{1}W_{2}\cdots W_{n-1}$, which can make the generated text meet the constraints of semantic and grammatical rules. This provides a redundancy for generative information hiding. The generation-based steganography method does not need to prepare the steganographic carrier in advance, but it directly generates text with smooth semantics, complete structure and natural appearance, and the secret information is embedded in the process of text generation.

The purpose of steganography is to hide the existence of information in the carrier and ensure the security of secret information. Therefore, security and embedding capacity are the primary evaluation criteria for steganographic systems. Perplexity (*ppl*) is usually used as the quality evaluation metric for generating steganographic text [14,24,25], as shown in Equation (2).

(2) $$\begin{array}{cc} ppl & =e^{-\frac{1}{N}\sum_{i=1}^{N}\ln P(S_{i})}\\ \; & =e^{-\frac{1}{N}\sum_{i=1}^{N}\ln P_{i}\left(W_{1},W_{2},\ldots ,W_{n}\right)} \end{array}$$

where *N* is the number of generated sentences, $s_{i}=\left\{W_{1},W_{2},\ldots W_{n}\right\}$ indicates the *i*-th sentence, and $P(S_{i})$ represents the probability distribution of words in $S_{i}$. Comparing Equation (2) with Equation (1), we find that perplexity is actually the difference in the statistical distribution of the language model between the generated text and the training text. The smaller its value, the more consistent the statistical distribution of the generated text with the training text.

Embedding capacity is usually measured by the Embedding Rate (*ER*), which is defined as the average number of secret bits that can be embedded per word (bpw), which is formalized as:

(3) $$ER=\frac{1}{N}\sum_{i=1}^{N}\frac{K_{i}}{L_{i}}$$

where *N* is the number of generated sentences, $K_{i}$ is the number of bits embedded in the *i*-th sentence, and $L_{i}$ is the length of the *i*-th sentence.

### 2.2. Linguistic Steganography Based on FLC

**Figure 1:**
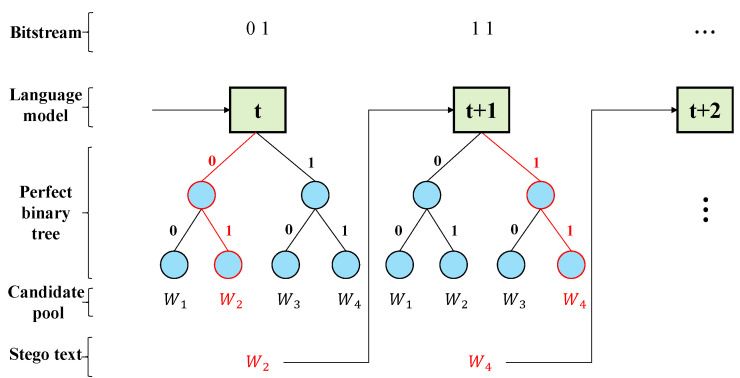
Fixed-length coding (FLC) proposed by [16].

Yang et al. [16] put forward two coding methods of FLC and VLC. Firstly, candidate words are arranged in descending order according to conditional probability. Then, the first *K* candidate words are selected to construct the candidate pool, and each word is coded by constructing a perfect binary tree or Huffman tree. A schematic diagram of FLC is shown in Figure 1. In this way, the embedding rate can be dynamically adjusted through the setting of the *K* value, so as to adapt to the differences in the demand for hidden capacity and concealment in different scenarios. The advantage of FLC is that the encoding and decoding are simple and fast, and the code length of each word is determined. The size of the candidate pool of FLC has a direct correspondence with the height *H* of the perfect binary tree: CPS = $2^{H}$. When the perfect binary tree is determined, then the coding of each candidate word is determined accordingly, and the code length of each word is also *H*. After that, the candidate word is selected for output according to the secret message to be embedded; thus, this word carries *H* bits of secret message.

This scheme determines one word per time step, and the secret message greatly interferes with the text generation process. To minimize the perplexity of the generated text, the conditional probability of the word selected at each time step should be as large as possible; however, due to the influence of the secret message to be embedded, the selected word may not be the most probable, which affects the quality of the generated text. Based on this, we propose a multi-time-step-based steganography scheme to select the optimal candidate word combination in multiple time dimensions. The specific scheme details will be described in Section 3.

## 3. MTS-Stega Methodology

In this section, we first introduce the overall framework of MTS-stega, then show the information hiding algorithm and information extraction algorithm, respectively, and finally perform theoretical analysis on the embedding rate, imperceptibility and robustness of this scheme.

### 3.1. Overall Architecture

Since our scheme uses *L* consecutive time steps to carry one unit of secret message *m*, we need to generate all the candidate words at *L* moments and select the optimal combination from them for output, instead of determining one word in one time step as in the traditional scheme. In this scheme, we choose a perfect binary tree with tree height *H* to encode the candidate words, so CPS = $2^{H}$, and the length of each word’s codeword is *H*.

**Figure 2:**
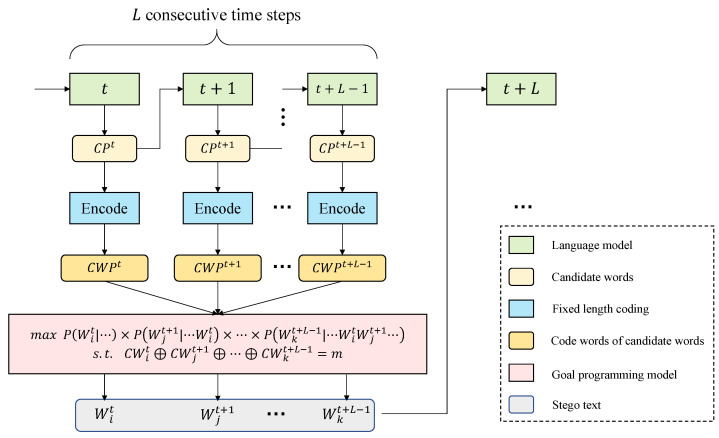
Overall framework of the proposed scheme.

Figure 2 outlines the overall framework of our scheme. First, we input the generated text into LM to obtain all candidate words and their conditional probability distributions at time *t*. The top $2^{H}$ words are selected in descending order of probability to form the candidate pool $CP^{t}$ at time *t*. After that, the $2^{H}$ words at time *t* are input into LM to obtain the probability distribution of words at time t+1. It is worth noting that each word in $CP^{t}$ corresponds to a set of candidate words at time t+1. We arrange each group of words in descending order of probability and take the top $2^{H}$ words to form $2^{H}$ basic candidate pools. We expand the concept of candidate pool and refer to these $2^{H}$ basic candidate pools collectively as the candidate pool at time t+1, and use $CP^{t+1}$ to refer to it—and so on until we obtain all candidate pools $CP^{t},CP^{t+1},\cdots ,CP^{t+L-1}$ for *L* time steps. After that, we encode the basic candidate pools in $CP^{t},CP^{t+1},\cdots ,CP^{t+L-1}$ using perfect binary tree, $W_{i}^{t}$ denotes the *i*-th candidate word in $CP^{t}$, and $CW_{i}^{t}$ is the codeword of $W_{i}^{t}$ with length *H*. Then, we obtain the set of codewords $CWP^{t},CWP^{t+1},\cdots ,CWP^{t+L-1}$ corresponding to the candidate words at *L* moments.

After obtaining the codewords of all candidate words, we find the combination of *L* candidate words $W_{i}^{t},W_{j}^{t+1},\cdots ,W_{k}^{t+L-1}$ satisfying the conditions as the output of these *L* time steps and also as the input of the next *L* time steps by solving the goal programming model. We describe the goal programming model in detail as shown in Equation (4).

(4) $$\begin{array}{cccc} \; & max & & P\left(W_{i}^{t}|\cdots \right)\times P\left(W_{j}^{t+1}|\cdots W_{i}^{t}\right)\times \cdots \times P\left(W_{k}^{t+L-1}|\cdots W_{i}^{t}W_{j}^{t+1}\cdots \right)\\ \; & s.t. & & CW_{i}^{t}⊕CW_{j}^{t+1}⊕\cdots ⊕CW_{k}^{t+L-1}=m \end{array}$$

where $P(W_{i}^{t}|\cdots )$ represents the conditional probability of the candidate word $W_{i}^{t}$ when the previous words are determined. According to Equations (1) and (2), the perplexity of the text generated by LM is related to the conditional probability of each word. The larger the product of the conditional probabilities of all words, the smaller the perplexity and the higher the quality of the generated text. The goal in the goal programming model is to maximize the conditional probability product of *L* consecutive words so as to reduce the overall perplexity of generated text. The constraint of the goal programming model is $CW_{i}^{t}⊕CW_{j}^{t+1}⊕\cdots ⊕CW_{k}^{t+L-1}=m$, which maps the binary secret information to the word space by *L* words to carry one unit of secret information *m*, which guarantees the correctness upon extraction. When we obtain the optimal combination of candidate words at this *L* time step, we add it to the generated stego text and input the model to embed the remaining secret message.

### 3.2. Information-Hiding Algorithm

The core idea of the information-hiding algorithm of this scheme is to use perfect binary tree coding to realize the mapping of secret messages to the word space, so that each *L* word in the steganographic text can carry a unit of secret message *m*, and the length of *m* is equal to the height of the perfect binary tree. To make the generated text more diverse, we first feed the introductory context into the model, on which we can condition the language model. The detailed process of information hiding is described in Algorithm 1.
**Algorithm 1** Information-Hiding Algorithm.**Input**: Secret bitstream M={0,0,1,0,1,⋯,0,1,0}; height of perfect binary tree *H*; introductory context; length of time step *L*; language model LM.**Output**: Generated steganographic text *C*.**Step 1:** Feed introductory context into LM to begin the generation process.**Step 2:** Calculate the probability distribution of the candidate pool ${CP}^{t}=\left[W_{1}^{t},\;W_{2}^{t},\cdots ,\;W_{2^{H}}^{t}\right]$ (*t* represents the *t* time step), sort it in descending order and limit its size by $2^{H}$.**Step 3:** For each candidate word $W_{i}^{t}$ ($i=1,2\ldots ,2^{H}$) in ${CP}^{t}$, feed $W_{i}^{t}$ into LM to obtain ${CP}^{t+1}$ for the next time step, and so on, until it is the t+L−1 time step; then, we can obtain ${CP}^{t},{CP}^{t+1},\cdots ,{CP}^{t+L-1}$.**Step 4:** One unit of *M* to be embedded is *m*, which has *H* bits. Based on the conditional probability distribution of each candidate pool for these consecutive *L* time steps, code the words by perfect binary tree (each word has a codeword length of *H*).**Step 5:** XOR the codes of all corresponding candidate word combinations and multiply the conditional probabilities. The candidate word combination (*L* words) with the greatest product of the conditional probabilities and whose XOR result is equal to *m* is selected and added to the generated steganographic text.**Step 6:** Repeat steps 2–5 until *M* is completely hidden.**Step 7:** Output the steganographic text *C*.

*H* and *L* in the input are the two hyperparameters of this scheme. *H* is the tree height of the perfect binary tree used for encoding. The larger *H* is, the longer the code length of each codeword is, and the more secret information it can carry. However, as *H* increases, the candidate pool will also become larger, which may cause some words with relatively low probability to be selected, thereby reducing the quality of the generated text. *L* represents that *L* time steps are used to carry one unit of secret information. The larger *L* is, the lower the embedding rate of the scheme will be, but there will be more candidate word combinations that meet the constraints of the goal programming model, so it is easier to obtain a good combination of candidate words to improve the quality of the text. However, in this case, the more time steps that need to be considered overall, the greater the amount of computation. When L=1, this scheme degenerates into an FLC scheme. Therefore, the selection of *H* and *L* needs to weigh the embedding rate, text quality and calculation amount. In practical applications, we usually take L=2, 1≤H≤6.

In step 1, we first input the introductory context to the model to constrain the text generated by the model later. This is to enable the generated stego text to adapt to different scenarios and meet different context needs.

In steps 3 and 4, we encode the candidate pools for *L* time steps. Although we have expanded the concept of the candidate pool and use all the basic candidate pools obtained by inputting words from the previous time step into LM as the expanded candidate pools for this time step, the size of the basic candidate pool is still $2^{H}$. For example, if there are $2^{H}$ candidate words in the candidate pool at time *t*, then after inputting these words into LM, we can obtain $2^{H}$ basic candidate pools with the size of $2^{H}$ for the next time step, and each word at moment *t* corresponds to one basic candidate pool at time t+1, and we encode for each basic candidate pool separately. So, the codeword length of each word in each basic candidate pool is *H* bits, which provides the basis for the constraints of the goal programming model that there exists *L* codewords whose XOR result can be equal to the secret message of *L* bits.

Step 5 is the concrete realization of the goal programming model of this scheme. The secret information is carried by the XOR result of the codewords of candidate words, and the perplexity of text is reduced by selecting the combination of words with the largest multiplication of conditional probability.

### 3.3. Information Extraction Algorithm

The method of information extraction uses encoding for mapping word space to binary bits during text generation. The receiver uses the same language model as the sender, obtains the probability distribution of the next word based on the initial input, encodes each candidate word based on a perfect binary tree, and extracts the code of corresponding candidate word based on the actual word selected for the steganographic text. Then, it XORs the extracted *L* codewords every *L* cycles to obtain a unit of secret message. Unlike the information-hiding algorithm, the information extraction algorithm does not need to input all the candidate words of the previous time step into LM each time to obtain the candidate pool for the next time step. Since the steganographic text is determined, we can directly determine the selected candidate word each time step and use it as the input for the next time step. The specific implementation details are described in Algorithm 2.
**Algorithm 2** Information Extraction Algorithm.**Input**: Steganographic text *C*; height of perfect binary tree *H*; introductory context; length of time step *L*; language model LM.**Output**: Secret bitstream M={0,0,1,0,1,⋯,0,1,0}.**Step 1:** Feed introductory context into LM to begin the extraction process.**Step 2:** Calculate the probability distribution of the candidate pool $CP=[W_{1},W_{2},\cdots ,W_{2^{H}}]$, sort it in descending order and limit its size by $2^{H}$.**Step 3:** Code each word $W_{i}$ in a perfect binary tree based on their conditional probability. Based on the actual accepted word in *C*, extract *H* bits codeword. Repeat steps 2–3 for *L* times.**Step 4:** XOR *L* codewords obtained in step 3, then add the *L* bits secret message to *M*.**Step 5:** Repeat steps 2–4 until *C* is completely processed.**Step 6:** Output extracted secret bitstream *M*.

Due to the characteristics of text itself, it will not be compressed or distorted during transmission like images or videos, so it has strong robustness, which makes the application scenarios of generation-based text steganography very extensive. For example, the transmission of stego text through instant messaging software such as Telegram and Skype, or the release of stego text through social media platforms such as Twitter and Facebook, can complete the concealed transmission of secret information. Then, the receiver can obtain stego text through browsing and copying from the platforms mentioned above and then extract the secret information from the stego text using our information extraction algorithm.

### 3.4. Comparative Analysis with Existing Methods

We combine the existing steganography schemes based on fixed-length coding to analyze the embedding rate and text quality of the proposed scheme MTS.

Fang et al. [14] (Bins) first split the vocabulary into $2^{B}$ blocks, each of which can be indexed with *B* bits. In the generation process, they select a word in the corresponding block for output according to the secret message of *B* bits each time step, so the embedding rate is *B* bits/word. FLC [16] performs perfect binary tree coding on the candidate pool of each time step, the tree height *H* is the length of a codeword, and it selects a candidate word for output according to the secret message of *H* bits to be embedded each time step, so the embedding rate is *H* bits/word. The proposed MTS performs perfect binary tree coding on the candidate pools of *L* consecutive time steps. The length of each codeword is the same as that of FLC, and the tree height is *H*, but we choose *L* codewords to carry one unit of secret message together, so the embedding rate is $\frac{H}{L}$ bits/word.

When B=H, Bins has the same embedding rate as FLC. Since FLC and MTS first sort the candidate words in descending order of conditional probability and select the first $2^{H}$ words for coding, the conditional probability of these words is relatively large. However, Bins does not consider the probability of word occurrence when dividing the dictionary, so the selected word may have a small probability, which affects the quality of the text. As ER increases, the number of blocks divided by the dictionary also increases. In some iterations, it may even be difficult to find a suitable word in the corresponding block as output, which makes the quality of the text generated by Bins drop rapidly as ER increases.

The embedding rate of FLC and MTS is closely related to CPS. The larger the CPS, the greater the ER, but the quality of the generated text will also decrease. This is because CP is sorted and truncated in descending order of conditional probability. When CPS increases, candidate words with low probability will appear in CP, which will lead to the possibility of selecting words with small probability based on secret information. The selection of each word in the FLC scheme depends on the secret information to be embedded each time step, so it is possible to select a word with a lower probability in CP, thus increasing the perplexity of generated text. However, the word combination selected by MTS among *L* consecutive time steps is the optimal probability combination under the constraints of the goal programming model, which can minimize the local perplexity of the text. The accumulation of this advantage can significantly reduce the perplexity of the generated text and improve the text quality compared to the FLC scheme.

### 3.5. Robustness Analysis

The traditional steganography methods assume that the carrier is transmitted without loss, so the receiver can extract the secret information completely without error. However, when a secret carrier is transmitted on a public channel, information is likely to be lost due to channel noise. For example, social networking platforms (such as Facebook, WeChat, etc.) will perform lossy processing on uploaded images and video carriers to save memory and bandwidth [26,27]. Due to the change of the secret carrier, the receiver cannot accurately extract the secret information, so the requirement of information integrity cannot be met. Therefore, steganography methods that use public channels such as social network platforms as covert communication channels need to consider both detection resistance and robustness. When the text is transmitted in the public network environment, almost no information is lost due to channel noise, so the hidden information it contains can retain a strong enough anti-interference ability. Therefore, text steganography has a natural advantage in robustness compared with schemes based on other carriers.

However, since generative text steganography uses the language model to embed secret information in the text generation process, the process of text generation also needs to be repeated during extraction. If one or more words in the stego text are modified or deleted, it will lead to a certain moment in which the corresponding word cannot be found in the candidate pool, which affects the subsequent extraction of secret information. The development focus of the existing generative text steganography schemes is to improve the text quality and semantic coherence of stego text, and it does not consider the problem of how to effectively extract secret information after the stego text is destroyed. We think this is an urgent problem to be solved in the future.

## 4. Experiments and Analysis

In this section, we evaluate the performance of MTS in terms of imperceptibility, embedding capacity and information extraction efficiency. Details of our experiments and the analysis of the results are present in the following subsections.

### 4.1. Experimental Setup

**Datasets.** We evaluated the performance of MTS on three public corpora, including “A Million News Headlines” (https://www.kaggle.com/datasets/therohk/million-headlines, accessed on 20 April 2022), “Microsoft Coco” [28], and “Movie Review” [29]. “A Million News Headlines” contains data on news headlines published by the Australian news source ABC (Australian Broadcasting Corporation) over an eighteen-year period, which contains 1,226,259 sentences. The average length of news headlines is 6 to 7 words. “Microsoft Coco” (MSCOCO 2017) is a large dataset published by Microsoft for object detection, segmentation and captioning. We selected the portion of the dataset used for image captions as our corpus, which contains 591,753 sentences. Most of these descriptive sentences are of medium length (about 10 words). The sentences are simple in structure and mainly describe people, objects and scenes. “Movie Review” (IMDB) mostly has long sentences (about 25 words), and the text is relatively diversiform, involving a variety of subjects. We randomly select 100 sentences from these three datasets for experiments (The sentences we used are available in https://github.com/yuxiaoxiaochun/MTS-stega, accessed on 20 April 2022, and the video of a real-time example is available in https://github.com/yuxiaoxiaochun/MTS-stega/releases/tag/real_time_example_vedio, accessed on 20 April 2022), and the statistics are shown in Table 2.

**Baselines.** We rebuilt Fang et al. [14] (Bins) and the FLC and VLC of Yang et al. [16] as baselines. For fair comparison, we rebuilt all the baselines with the same language model, which is the 345M parameter GPT-2 model [30].

### 4.2. Imperceptibility Results

The purpose of a concealment system is to hide the existence of information in the carrier to ensure the security of important information. Therefore, the imperceptibility of information is the most important performance evaluation factor of a concealment system.

Since Bins is coded according to blocks, the coding length of each block is the same. During the steganography process, the corresponding block is retrieved according to the secret information, and a word is selected from it. So, we can migrate the concept of the candidate pool to Bins, and the size of the candidate pool is just the number of divided blocks.

**Figure 3:**
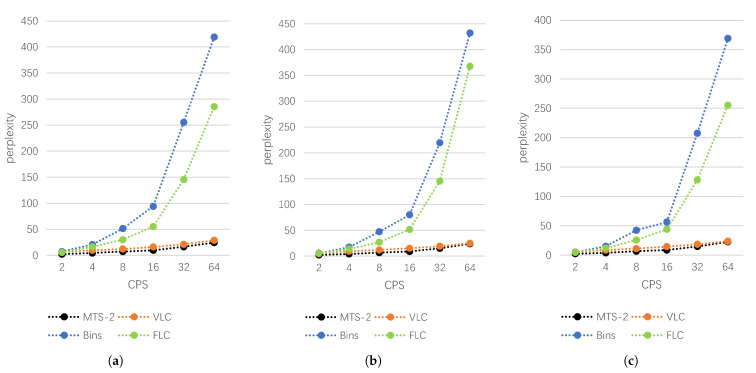
The results of different steganography methods at different CPS on each dataset. (**a**) News Headlines, (**b**) MSCOCO, (**c**) IMDB.

We take each text in the three datasets as confidential information, first convert the confidential text into secret bitstream, and then use Bins, FLC, VLC and the proposed scheme to generate steganographic texts, respectively, when CPS = 2, 4, 8, 16, 32, and 64. In all experiments, we choose L=2 and call it MTS-2. In order to ensure the diversity of generated steganographic text, before generating text, we input the text in each dataset as introductory context to LM. The experimental results are shown in Table 3. For a more intuitive display, we have drawn line charts on the three datasets, respectively, as shown in Figure 3.

Based on these results, we can draw the following conclusions. First, on each dataset, for each steganography algorithm, the perplexity gradually increases as CPS increases. That is, the statistical linguistic distribution difference between the generated text and the training samples will gradually increase. This is because as the number of embedding bits per word increases, during each iteration, the word selected as the output is more and more controlled by the number of embedding bits, making it increasingly difficult to select the word that best fits the statistical distribution of the training text. Secondly, the quality of steganographic text generated by the variable-length coding scheme is higher than that of the fixed-length coding schemes under the same CPS. This is because the variable-length coding makes the codeword length of the word with a larger conditional probability in the candidate pool shorter, and it has a greater probability of being selected for each time step, but because the codeword becomes shorter, the secret information carried by a word will be less, and the embedding rate will be smaller than the fixed-length coding scheme under the same CPS. Compared with other schemes, MTS-2 has an obvious advantages in the quality of generated text, which is even better than the variable-length coding scheme. This is due to the trade-off of MTS-2 over two time steps. Compared with other schemes, the choice of words is more flexible, and it is easier to select the word combination with the largest multiplication of conditional probability.

**Figure 4:**
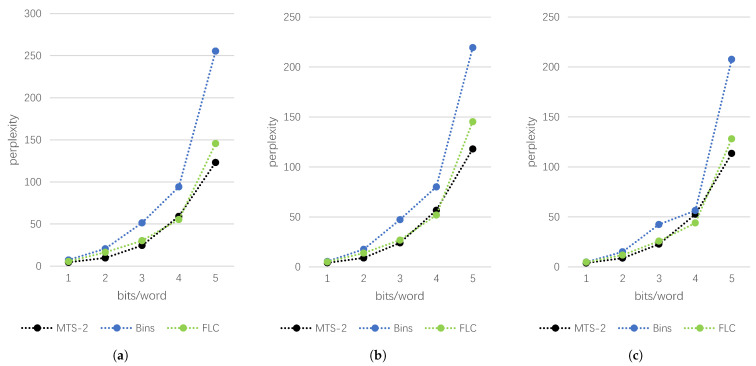
The results of different steganography methods at different ER on each dataset. (**a**) News Headlines, (**b**) MSCOCO, (**c**) IMDB.

Since MTS-2 utilizes two words to carry one unit of secret information, the embedding rate is half of Bins and VLC under the same CPS. Next, we analyze the text quality of each scheme under the same embedding rate. Since the embedding rate of VLC is uncertain for each secret text and has no direct correspondence with CPS, we do not compare it this time. The experimental results are shown in Table 4, and the line graph is shown in Figure 4.

Based on the above results, we can know that although the embedding rate of MTS-2 is smaller than that of Bins and FLC under the same CPS, the embedding rate of MTS-2 can be improved by increasing the height of the perfect binary tree and the length of each codeword, while still maintaining a high text quality.

Table 5 shows two steganographic examples of MTS-2 in the case of ER = 3 bits/word and ER = 4 bits/word, respectively.

### 4.3. Results of Information Extracting Efficiency

**Figure 5:**
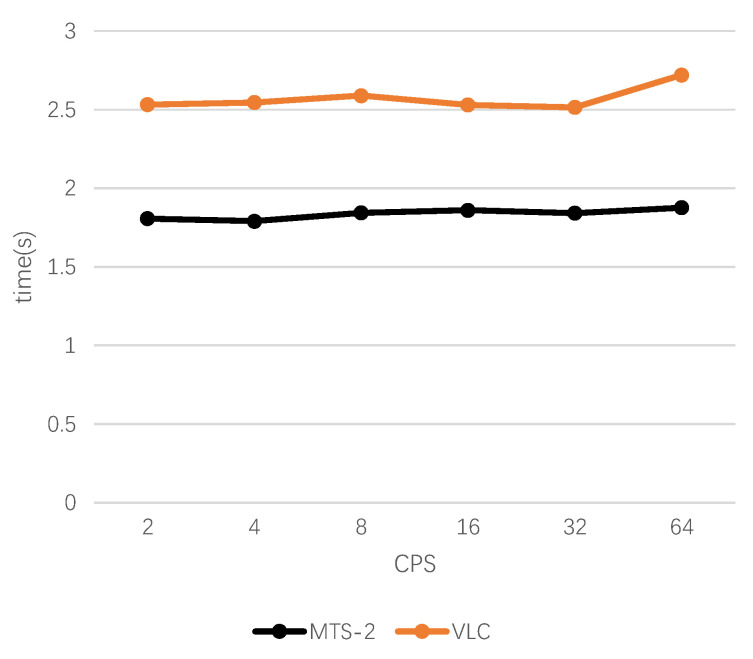
Mean extraction time of VLC and MTS-2 when the steganographic texts have the same length.

For the steganographic schemes, since the words in the candidate pool need to be tree-coded at each iteration, the size of the candidate pool will significantly affect the efficiency of information extraction. The proposed scheme selects the perfect binary tree to encode the candidate words and inherits the decoding advantages of the fixed-length coding scheme. We selected 100 long texts from the datasets as secret messages and limited the length of the generated steganographic texts to 100 words. We use VLC and MTS-2 to conduct experiments according to the guidance of [31] and record the information extraction time at CPS = 2, 4, 8, 16, 32, 64. The results are shown in Table 6 and Figure 5.

It can be seen that the proposed scheme has higher decoding efficiency than VLC, and with the increase of CPS, the decoding time does not change significantly. This is because the VLC scheme uses a Huffman tree to encode candidate words, and the time complexity of constructing a Huffman tree is O(nlogn), which is higher than O(n) for constructing a perfect binary tree. With the increase of CPS, the tree depth increases, and the construction of the Huffman tree will consume more time. In the MTS-2 scheme, since the words in the candidate pool are already arranged in descending order of the conditional probability, and CPS = $2^{H}$, it is not even necessary to construct a perfect binary tree in the specific implementation, but the codeword of each candidate word can be directly determined, so the decoding efficiency of MTS-2 is higher than that of VLC.

### 4.4. Experimental Summary

In this section, we compared the proposed scheme with Bins, FLC and VLC in terms of concealment, embedding capacity and information extraction efficiency. The experimental results show that MTS-2 has the smallest text perplexity under the same CPS; the quality of the steganographic text under the same embedding rate has obvious advantages over the fixed-length coding schemes Bins and FLC; the information extraction efficiency is significantly better than the variable-length coding scheme VLC.

Since the proposed scheme uses *L* words to carry one unit of secret message, the text length is *L* times as long as Bins and FLC with the same ER, and we need to consider the candidate words of *L* time steps, so these will undoubtedly increase a lot of computation when generating text. These are the costs of improving the quality of steganographic text. In our experiments, we found that when L=2, the purpose of reducing the perplexity of steganographic texts can be well achieved, and the amount of computation in the information-hiding stage is relatively low, and it has achieved good results in terms of steganographic text quality and computation amount. Therefore, only the experimental results of MTS-2 are shown in this section.

## 5. Conclusions

In this paper, we propose a linguistic steganography scheme based on multi-time-step by taking advantage of the decoding superiority of fixed-length coding and addressing the shortcomings of conventional generative text steganography schemes. We trade off multiple time steps in generating text using language model, utilize multiple words to carry a unit of secret information, and select the optimal combination of candidate words by solving the goal programming model to effectively improve the quality of steganographic text. The experiments verify the advantages of this scheme in terms of generated text quality and information extraction efficiency. Meanwhile, there are still some deficiencies in the proposed scheme, which need to be solved in our future work.

- Since the calculation amount of our scheme when generating steganographic text increases with the increase of *L*, how to reduce the calculation amount when *L* is relatively large is an issue that requires further research.
- Due to the limitations of generative text steganography, existing schemes cannot resist attacks such as word modification or deletion, which is an urgent problem to be solved.
- The proposed scheme can generate coherent and high-quality stego text but cannot effectively control its subject or emotion and other attributes, so it is not applicable in some scenarios that require precise control of semantic attributes. This is also our future direction.

## Figures and Tables

**Table 1:** Notations, abbreviations and descriptions.

Notations	Descriptions
LM	language model
ppl	perplexity, an evaluation metric for the quality of generated text
ER	embedding rate, the average number of secret bits that can be embedded per word
FLC	fix-length coding based on perfect binary tree proposed by [16]
VLC	variable-length coding based on Huffman tree proposed by [16]
*t*	time step in the process of LM-generating text
*CP* (${CP}^{t}$)	candidate pool (in time step *t*), consisting of candidate words
CPS	the size of a candidate pool
*H*	the height of a perfect binary tree
*L*	how many consecutive time steps used to carry a unit of secret message, *L* can be 2,3,4,⋯, and the scheme using *L* can be called MTS-*L*
$W_{i}^{t}$	the *i*-th word in $CP^{t}$
$CW_{i}^{t}$	codeword of $W_{i}^{t}$ after fix-length coding

**Table 2:** Datasets statistics.

Dataset	News Headlines	MSCOCO	IMDB
Num. of Sentences	100	100	100
Avg. Num. of Words	6.44	10.44	25.77

**Table 3:** The mean of the perplexity results of different steganographic methods under the same CPS.

Dataset	CPS	Bins [14]	FLC [16]	VLC [16]	MTS-2
News headlines	2	7.3523	5.6029	6.608	**2.3645**
	4	20.6893	16.3785	9.8417	**4.5797**
	8	51.4391	30.233	12.2796	**7.3124**
	16	94.1353	55.3581	16.1114	**9.7485**
	32	255.3615	145.5817	21.2936	**16.5474**
	64	418.8856	285.5139	28.8719	**24.5283**
MSCOCO	2	5.554	5.1126	5.8332	**2.2973**
	4	17.578	13.8499	9.4598	**4.1502**
	8	47.3078	26.9079	12.0263	**6.8475**
	16	80.1382	51.9122	15.2561	**9.0102**
	32	219.519	145.321	19.1511	**15.0822**
	64	432.0135	367.5067	24.9883	**24.0809**
IMDB	2	4.9013	4.7677	5.215	**2.4626**
	4	15.1401	11.8964	8.7275	**3.9473**
	8	42.4247	25.8169	11.22	**6.7513**
	16	56.3714	43.9763	14.5684	**8.7607**
	32	207.6012	128.1702	18.4876	**14.8658**
	64	368.9476	255.3289	23.8892	**22.7323**

**Table 4:** The mean of the perplexity results of different fixed-length coding-based steganographic methods under the same embedding rate.

Dataset	ER (Bits/Word)	Bins [14]	FLC [16]	MTS-2
News Headlines	1	7.3523	5.6029	**4.5797**
	2	20.6893	16.3785	**9.7485**
	3	51.4391	30.233	**24.5283**
	4	94.1353	**55.3581**	59.1203
	5	255.3615	145.5817	**123.1296**
MSCOCO	1	5.554	5.1126	**4.1502**
	2	17.578	13.8499	**9.0102**
	3	47.3078	26.9079	**24.0809**
	4	80.1382	**51.9122**	56.7739
	5	219.519	145.321	**118.0109**
IMDB	1	4.9013	4.7677	**3.9473**
	2	15.1401	11.8964	**8.7607**
	3	42.4247	25.8169	**22.7323**
	4	56.3714	**43.9763**	52.6528
	5	207.6012	128.1702	**113.5318**

**Table 5:** Steganography example by MTS-2.

Introductory context	The lives of several individuals intertwine as they go about their lives in their own unique ways, engaging in acts society as a whole might find disturbing in a desperate search for human connection.
Secret message 1	Eve is not who she says she is.
Steganographic text 1 (ER = 3 bits/word)	There is no shame in using music to explore the possibilities of existence. Though the use of music as a bridge between communities and reality can be seen as positive, it sometimes becomes a temptation to put too much baggage such as emotional baggage such as family, job, education, environment, and religious beliefs out of its light. So, does the music tell us some good things about what some people know about people? While it
Secret message 2	The secret code is X3SJ83F.
Steganographic text 2 (ER = 4 bits/word)	Children are subject to dehumanization, trauma, harm, suicide, intimidation, pressure, deprivation, injustice, rape, repression, violent demonstrations, coercion, racism, institutionalized inhumanity, psychological abuses, and myriad other methods of everyday control. Whether it’s threats of detention

**Table 6:** The results of the mean extraction time of this scheme and the variable-length coding steganography scheme under the same CPS.

CPS	VLC [16] (s)	MTS-2 (s)
2	2.5314	**1.8066**
4	2.5452	**1.7904**
8	2.589	**1.8429**
16	2.5294	**1.8602**
32	2.5141	**1.8413**
64	2.7205	**1.8766**

## Data Availability

Not applicable.
